# Parent Artery Disease-Related Stroke: What Is the Impact on Endovascular Treatment? A Narrative Review

**DOI:** 10.3390/jcm15030983

**Published:** 2026-01-26

**Authors:** Marialuisa Zedde, Francesca Romana Pezzella, Piergiorgio Lochner, Rosario Pascarella

**Affiliations:** 1 Neurology Unit, Stroke Unit, Azienda Unità Sanitaria Locale-IRCCS di Reggio Emilia, Viale Risorgimento 80, 42123 Reggio Emilia, Italy; 2 Neurosciences Department, Azienda Ospedaliera San Camillo Forlanini, 00152 Rome, Italy; 3 Department of Neurology, Saarland University Medical Center, 66421 Homburg, Germany; 4 Neuroradiology Unit, Ospedale Santa Maria della Misericordia, AULSS5 Polesana, 45100 Rovigo, Italy

**Keywords:** parent artery disease, PAD, intracranial stenting, stroke, atherosclerosis, intracranial stenosis, MRA, DSA

## Abstract

**Background/Objectives:** Parent artery disease (PAD) is a significant yet often overlooked contributor to ischemic strokes, particularly affecting the perforating arteries. This study aims to evaluate the impact of PAD on endovascular treatment outcomes in patients with intracranial atherosclerosis. **Methods:** A narrative review was conducted, synthesizing the existing literature on PAD and its relationship with endovascular interventions. Key studies were analyzed to assess the effectiveness of imaging techniques like high-resolution Magnetic Resonance Imaging (MRI) and the implications of plaque morphology on treatment strategies. **Results:** The findings indicate that PAD significantly complicates endovascular procedures, often leading to perforating artery occlusions and increased rates of stroke recurrence. Patients with PAD-related strokes demonstrated larger lesion volumes and more severe neurological deficits compared to those with small vessel disease. The review highlights the challenges of accurately diagnosing PAD using conventional imaging techniques, emphasizing the need for advanced modalities to identify atheromatous plaques that may not cause significant stenosis. **Conclusions:** The study underscores the necessity for a shift in clinical practice towards recognizing and managing PAD in patients with ischemic strokes. Enhanced imaging techniques and tailored endovascular strategies are essential to improve patient outcomes and minimize the risk of recurrent strokes. Further research is needed to establish comprehensive guidelines for addressing PAD in acute stroke management.

## 1. Introduction

The term “parent artery disease” (PAD), also known as “branch occlusive disease” (BOD), refers to the involvement of the ostia in perforating arterioles through the steno-occlusion of the parent artery, including non-stenosing atheromas. A parent artery was defined as an original artery that branches out and forms a small artery, which is responsible for index small subcortical infarction (SSI) [1,2]. Usually, atherosclerosis is the most prevalent cause of this disease, but even acute changes in the plaque (e.g., thrombus on a complicated plaque) and occlusion from other causes against an atheromatous background can produce this pattern. The corresponding lesion has a lacunar phenotype on imaging and it usually involve multiple sequential perforating arteries, resulting in a slightly greater size than the lacunar stroke related to small vessel disease (SVD). Sometimes, it is difficult to identify non-stenosing plaques in the parent arteries due to vascular remodeling and the Glagov phenomenon [3]. In fact, even mild stenosis (<50%) may result in PAD without changing the luminal profile of the artery in angiographic imaging [3]. PAD-related SSI has been widely described, initially in the Asian population and finally in the Western population [3].

The impact of PAD on the endovascular treatment of patients with intracranial stenosis and plaque-induced thrombotic occlusion has been discussed but has not been the subject of dedicated studies. Although the topic is relevant, solid evidence is lacking, making a systematic review approach inapplicable. The goal of this narrative review is therefore to address the issue comprehensively, presenting the available information on this topic and drawing conclusions for clinical practice in terms of both diagnosis and acute treatment. The terms PAD and BOD are used interchangeably in the following sections. The paucity of data about the role of PAD in the endovascular treatment of intracranial stenosis prevents a systematic approach. We also performed an extensive, but not systematic, research in English-language published papers in the last 25 years, identified the few most informative papers about perforating artery issues in the endovascular treatment of patients with ischemic stroke and extended the research to papers citing the previously identified studies. After these steps, we provided a narrative summary and interpretation of the main issues addressed by these papers.

## 2. Definition and Burden

### 2.1. Burden of Intracranial Atherosclerosis

Intracranial atherosclerosis, commonly referred to as intracranial atherosclerotic disease (ICAD), is a progressive condition marked by the formation, evolution, and complications of atherosclerotic plaques in major intracranial arteries [4]. It is recognized as a leading cause of ischemic stroke, particularly among Asian populations, and is also prevalent in Hispanic and African communities [5,6,7]. In Caucasians, it accounts for approximately 10% of ischemic stroke cases, although studies suggest that the prevalence of intracranial atherosclerotic plaques may be greater than previously proposed [2,8,9]. Consequently, ICAD is likely a significant contributor to stroke and vascular cognitive impairment globally [10].

An autopsy study involving 339 Caucasian patients with stroke revealed that intracranial plaques and stenoses were found in 62.2% (95% CI, 56.3 to 68.1) and 43.2% (95% CI, 37.2 to 49.3) of those with brain infarctions, respectively, in contrast to 48.8% (*p* < 0.05) and 17.5% (*p* < 0.001) of patients with brain hemorrhage. Notably, in 43% of patients with brain infarctions exhibiting at least one intracranial plaque (luminal stenosis < 30%), an intracranial stenosis was deemed causative in 5.8% of cases due to clots superimposed on ulcerated plaques, with 27% of patients having stenosis levels between 30% and 75%. Multivariate analysis indicated significant associations between diabetes, male sex, and the presence of intracranial plaques and stenosis, as well as a history of myocardial infarction correlating with plaques, while prior strokes were linked to intracranial stenosis. Importantly, intracranial plaques were detectable in all etiological categories of stroke, highlighting a significant and often underestimated issue within the Western population. Moreover, a notable correlation exists between ischemic stroke and intracranial atheromatosis even when significant stenosis is absent, which points to the potential for positive remodeling of the affected arterial segments—a phenomenon known as the Glagov effect—thus raising concerns about the reliability of traditional luminographic methods in excluding intracranial atheromatosis with stenosis below 50%.

ICAD is an aggressive disease, with symptomatic patients facing an elevated risk of recurrent ischemic events despite optimal medical therapy, as evidenced by randomized controlled trials [11]. However, substantial uncertainty remains about the most effective treatment approaches for ICAD, especially in high-risk patients [12]. The growing use of cerebral vascular imaging techniques for therapeutic decision-making during the hyperacute phase of stroke has made the identification of ICAD possible across a broader patient population. Within this framework, ICAD can either be a direct cause of ischemic events or a coexisting condition whose prognostic significance—both in the acute phase and for future stroke risk assessment—remains inadequately understood [13]. However, the unique anatomical features of intracranial arteries may lead to distinct aspects of the atherosclerotic process potentially influencing clinical outcomes such as the hemodynamic effects of stenosis [14]. Lastly, advancements in vessel-wall imaging techniques have shifted our focus from merely evaluating intracranial stenosis to assessing atherosclerotic plaques, allowing for a comprehensive examination of all stages of ICAD, including early subclinical phases, which presents a significant opportunity to enhance primary stroke prevention [15,16].

Typically, ICAD refers to atherosclerotic plaques affecting major intracranial arteries across various stages of the disease, including non-stenotic forms. Conversely, the term intracranial stenosis (ICAS) is reserved for situations where plaques cause significant luminal narrowing (generally exceeding 50%) [17,18]. Severe arterial caliber reductions (greater than 70%) and/or associated hemodynamic compromise are classified as high-grade intracranial stenosis. This hemodynamic compromise is characterized by a notable reduction in anterograde blood flow in the downstream arterial territory, which subsequently activates collateral circulation to maintain perfusion to brain tissue and enhance embolic clearance in distal arteries [17,18]. In cases of severe hemodynamic compromise, collateral circulation may be inadequate, leading to insufficient brain tissue perfusion and detectable hypoperfusion on perfusion imaging techniques. Clinical symptoms may also reflect hemodynamic compromise, particularly if patients exhibit fluctuating neurological deficits in response to postural changes, postprandial states, or drops in blood pressure. The patterns of infarcts observed through neuroimaging can serve as indirect indicators of hemodynamic compromise, especially in cases presenting with cortical or internal borderzone infarctions.

Diagnosing atherosclerosis as the underlying cause of stenosis can be challenging due to the myriad of conditions that may lead to intracranial arterial lumen reductions, particularly during the acute phase of ischemic stroke. Accurate differentiation from other intracranial arteriopathies, such as infectious or non-infectious vasculitis, dissections, reversible cerebral vasoconstriction, or arterial vasospasm, is crucial. In this context, high-resolution arterial-wall MRI is gaining importance for characterizing intracranial atherosclerotic plaques and distinguishing them from other causes of stenosis [19,20].

### 2.2. Perforating Arteries: Tips and Tricks

The anatomy and distribution of deep-perforating arteries within the brain are beyond the scope of this review; however, recent studies have addressed this topic and they can be referred to [21,22]. This review will primarily focus on the M1 segment of the middle cerebral artery (MCA) and the basilar artery (BA), along with their corresponding perforators, as these are crucial for endovascular approaches.

An area that warrants further investigation is the network of deep cerebral perforating arteries, such as MCA and BA [21,23,24]. Despite their small diameters (typically less than 1 mm) these arteries play a critical role in several cerebrovascular disorders, including lacunar infarcts, intracerebral hemorrhages, dementia, and complications arising from subarachnoid hemorrhages and neurovascular interventions [25,26,27,28,29,30]. The currently available imaging techniques can visualize only a limited portion of these arteries, which limits our understanding of their anatomical pathways and hemodynamics [31,32,33,34]. Additionally, animal models often fall short due to anatomical differences in the neurovascular systems [35,36,37].

Only a limited number of studies examined the geometry of perforating artery ostia. One such study [38] focused on the morphology of these first segments, which serve as connection points between high-pressure intracranial arteries and their microcirculation. In this study [38], twenty-three unfixed specimens from the human basal ganglia with MCA and ten specimens from the brainstem with BA were examined after filling the arteries with a barium sulfate contrast medium. The specimens were then fixed with a 10% buffered formalin solution and analyzed using a microtomography scanner (with a voxel size of approximately 25 μm). Cross-sections were created along the long axis of the MCA, BA, internal carotid artery (ICA), and vertebral artery, which included the ostia of the perforating arteries and smaller branches (such as the anterior inferior cerebellar artery and the anterior choroidal artery) for histopathological examination. The main finding was that the ostia of deep cerebral-perforating arteries are not round but rather ellipsoidal in shape, a result of local neointimal remodeling of the arterial wall [38]. Regarding each perforating artery, its initial segment (often very short) is oriented perpendicularly to the parent artery. According to Murray’s law [39,40], if the diameter of a branching artery is significantly smaller than that of the parent artery, then the angle between the branches is approximately 90°, leading to a round cross-section of the ostium. Even if the perforator has a recurrent parallel course, its initial segment remains perpendicular to the parent vessel. Conversely, if the elliptical shape arises from oblique branching, the long axis of the ostium is expected to align with the parent artery, which is often not the case. The orientation of the origins of perforating arteries closely correlates with their spatial relationships to the parent artery. When a perforating artery runs parallel to the parent vessel, the short axis of the ellipsoidal origin also aligns parallel to the parent artery; as the angle of the perforator deviates from that of the parent artery, the orientation of the ellipse rotates accordingly [38].

Extensive research on hemodynamic conditions around the origins of intercostal arteries reveals complex patterns of wall shear stress [41] and arterial remodeling in response to disturbed flow [42], providing useful information for other vascular beds. Stenoses at the ostia may function as pressure-reducers at the junctions between high-pressure intracranial arteries (such as the MCA and BA) and the perforating arteries, thereby adjusting blood pressure to suit the microcirculation [43]. The hemodynamic conditions are expected to be complex; the analogy with intercostal arteries may not be entirely applicable, as the origins of the perforating arteries in SVD are believed to be primarily influenced by atherosclerosis in the parent artery obstructing the perforator origins. This concept is based on case series involving patients with lacunar infarction [44,45,46,47]. The severity of ostial stenosis is linked to both age and the extent of atherosclerosis—an indicator of cardiovascular health—with ostia being observed to be narrower in older individuals and those with more severe atherosclerosis [38,44,45,46,47]. These findings imply an increase in narrowing with age and dependence on cardiovascular risk factors like hypertension. Notably, even young adults exhibited stenosed ostia [38]. This observation aligns with the proposed function of pressure-reducers: normal arterial blood pressure may be excessively high for perforating arteries, while elevated blood pressure, typical in older individuals and those with atherosclerosis, necessitates further reduction to accommodate microcirculation; thus, a more pronounced reducer is required [38]. This association was particularly noted in the subgroup of MCA perforators with a parallel course, which tend to exhibit more severe stenoses, suggesting that the ostia of perforating arteries can be affected by atherosclerosis. The presence of atherosclerosis in the wall of the parent artery from which the perforating vessel originates may exacerbate this issue, contributing to hypoperfusion or occlusion in the perforating artery itself [38].

### 2.3. Pathology of Intracranial Atherosclerosis

From a pathological perspective, atherosclerosis is characterized as a cholesterol-mediated disorder that leads to the formation of cholesterol deposits, creating atheromas within the arterial walls. This condition, often inferred from coronary artery disease, follows a simplified pathophysiological model that begins with endothelial dysfunction and the subsequent accumulation of cholesterol particles in the intima [48]. Circulating monocytes adhere to the activated endothelium and infiltrate the vessel wall, transforming into macrophages that bind to lipoprotein particles, thereby becoming foam cells. T lymphocytes also participate, to a lesser extent, in this process. In response to the infiltrating leukocytes, smooth muscle cells migrate from the tunica media into the intima, promoting collagen matrix synthesis that can lead to a pathological thickening of the intima [49]. The continued accumulation of lipids, leukocytes, and smooth muscle cells culminates in the formation of atheromas [50]. These inflammatory components not only contribute to plaque progression but can also trigger clinical events even in the absence of significant stenosis [51].

The classification of atheromatous lesions is primarily based on the characteristics of the fibrous cap, the extent of the lipid-rich necrotic core, and various complicating features such as thrombi [50]. The fibrous cap, formed through smooth muscle cell-mediated collagen synthesis, serves as a protective layer over the lipid core [52]. However, the inflammatory response can compromise the integrity of the fibrous cap, leading to its erosion and thinning, which heightens the risk of plaque rupture—a critical complication that can precipitate acute thrombosis [53]. Intraplaque hemorrhage and neovascularization within plaques are additional indicators of plaque vulnerability [54].

Hemodynamic environments characterized by low wall shear stress and circumferential wall tension significantly promote atherogenesis [55]. Low wall shear stress, often associated with recirculating and oscillatory flow patterns, occurs at arterial walls situated opposite to flow dividers or arterial branch points, where blood flow dynamics are altered [56]. The tension within the arterial walls compensates for these non-laminar flow patterns; however, this compensation may fail over time due to arterial stiffness from prolonged stress, particularly in hypertensive individuals [57]. In the intracranial circulation, atherosclerotic plaques are frequently found on the ventral wall of the MCA, opposite the origins of perforating arteries, as well as on the lateral walls of the proximal basilar artery, where the confluence angle of the vertebral arteries influences flow patterns [58].

In coronary arteries, atheromatous lesions typically expand outward, away from the lumen, preserving the luminal diameter [59]. However, in vivo imaging and pathological studies have revealed inconsistent findings regarding the outward remodeling of intracranial arteries in response to atheroma formation [60,61]. Distinct anatomical features of cerebral arteries may contribute to the unpredictable nature of intracranial outward remodeling, including the absence of an external elastic lamina, a relatively thicker media compared to coronary arteries, complex collateralization, and the autoregulatory capabilities of distal cerebral arterioles [62]. A deeper understanding of how intracranial arteries remodel could enhance plaque detection and risk assessments for ICAD, and inform future therapeutic strategies.

ICAD can lead to ischemic stroke through various mechanisms: vulnerable plaque rupture resulting in thrombosis that causes in situ occlusion or artery-to-artery embolism, hemodynamic failure from high-grade stenosis, or branch occlusive disease due to intimal impingement at the origins of small perforating arteries [63]. The latter may manifest even with mild luminal stenosis, presenting as lacunar infarctions [64]. While artery-to-artery embolism and branch occlusive disease may be more prevalent mechanisms, it is common for multiple mechanisms to coexist [65].

Each of these mechanisms carries distinct prognoses and risks of stroke recurrence, emphasizing the importance of identifying high-risk subgroups that may benefit from targeted treatment strategies [66]. Notably, ICAD exhibiting features of hemodynamic failure is associated with a particularly elevated risk of stroke recurrence [67].

### 2.4. PAD in Etiological Classifications of Ischemic Stroke

ICAD and PAD are frequently overlooked in the predominant classifications used for ischemic stroke etiology. The only classification that explicitly addresses this issue is the Chinese Ischemic Stroke Classification (CISS) [68]. In contrast, the most widely recognized system is the TOAST criteria [69], which has been enhanced by the SSS-TOAST [70,71] and Korean TOAST [72], both of which refine the diagnostic criteria for various atherosclerosis subtypes and small artery occlusions. The A–S–C–O and A–S–C–O–D classifications [73,74] are more applicable to secondary stroke prevention and clinical trial design, yet they are not commonly utilized in everyday practice, similarly to the Causative Stroke System (CSS) classification [75]. Notably, all these classification systems fail to consider intracranial atheromatous branch disease that affects penetrating arteries and do not delve into the underlying mechanisms of ischemic strokes attributed to large artery atherosclerosis (LAA). Specifically, PAD is recognized as a distinct subcategory in the CISS classification, defined as isolated penetrating artery territory infarction with evidence of atherosclerotic plaque (identified through high-resolution MRI) or any degree of stenosis in the parent artery.

The primary focus within this context is subacute small subcortical infarctions (SSIs), traditionally referred to as “lacunar infarctions.” These SSIs have typically been attributed to SVD, which is pathologically characterized by lipohyalinosis and fibrinoid degeneration [76,77,78]. However, atherosclerosis in the parent artery can also lead to SSI by obstructing the orifices of branch arteries, contributing to PAD [44]. This phenomenon is particularly prevalent in Asian populations, where intracranial atherosclerosis is more common. In the 1970s, Fisher reported three autopsy cases of infarctions in the paramedian pons caused by the occlusion of perforating branches of the BA. The pathological substrates responsible for occluding these perforators included atheroma, local hemorrhage, and microdissection, which contained histological components such as fatty macrophages, red blood cells, and fibrous plaques [46,47]. Subsequent reports also documented SSIs resulting from the occlusion of perforators due to focal atherosclerotic plaques in the M1 MCA [79,80]. These findings led to the coining of the term intracranial BOD, emphasizing that SSIs can arise from atheromatous rather than purely lipohyalinotic causes. Caplan classified BOD into three categories: (1) plaque from the parent artery obstructing the orifice of a branch artery; (2) plaque from the parent artery extending into the branch artery; and (3) obstruction of the proximal portion of the branch artery by microatheroma [44]. The development of atherosclerosis is influenced by factors such as flow velocity and wall shear stress [81,82], leading to higher incidences of atherosclerosis at arterial branching points, which can result in perforator occlusion.

### 2.5. PAD Burden

The advent of magnetic resonance angiography (MRA), computed tomographic angiography (CTA), and transcranial Doppler ultrasound has significantly facilitated the diagnosis of intracranial atherosclerosis, leading to more frequent identification of PAD-related SSIs [83]. This condition has been variably labeled as SSI with parent artery disease (SSI + PAD) [84], BOD [85,86], or local branch occlusion [87].

Arteriosclerotic SSIs typically affect the proximal segments of small vessels, resulting in relatively large infarcts that often involve multiple perforating arteries [44]. The size and extent of these SSIs are used to distinguish arteriosclerotic causes from SVD. For instance, BOD within the lenticulostriate artery territory is defined as an infarct larger than 10 mm in diameter, visible across three or more axial slices [88]. However, diffusion-weighted imaging (DWI) often shows that lesion volumes of SSIs can increase over time [89,90], indicating that the size of the lesions might vary depending on the timing of the imaging [88]. Although larger SSIs are more likely to stem from non-SVD causes [91], relying solely on lesion diameter is insufficient to differentiate between PAD and SVD as the underlying etiology [92]. Additionally, SSIs that are located far from their parent vessels can also be linked to PAD [84,93], suggesting that BOD cannot be accurately defined by MRI criteria alone.

PAD-related SSIs can occur across various arterial territories, including the lenticulostriate arteries of the MCA, pontine paramedian arteries of the BA, thalamic perforating arteries of the posterior cerebral artery (PCA), medullary arteries of the vertebral artery (VA), and branches of the anterior cerebral artery (ACA). Notably, PAD serves as a more significant stroke mechanism in the posterior circulation compared to the anterior circulation.

In the territory of the lenticulostriate artery, PAD-related SSIs typically present as vertical lesions extending from top to bottom along these arteries [94,95]. A study involving 102 Korean patients with small SSIs (less than 1.5 cm in diameter) identified MCA atherosclerosis as the cause of infarction in 27 patients (35%) [96]. Another MRA-based study in Korea reported that MCA atherosclerosis was implicated in 33 out of 118 patients (28%) with DWI-identified SSIs [92]. A study conducted in Hong Kong examined 226 patients who underwent DWI and MRA, finding that 71 had small SSIs (ranging from 0.2 to 2.0 cm in diameter), with 12 (16.9%) having associated intracranial atherosclerosis [97].

In another MRA-based study from Korea, PCA atherosclerosis was identified in 76 out of 205 patients (37%) with PCA territory infarctions [98]. The occlusion of thalamic perforators was the predominant mechanism for focal thalamic infarctions in patients with intrinsic PCA atherosclerosis [98]. In Asian populations, SSIs resulting from PCA atherosclerosis were observed in 7–22% of patients with focal lateral thalamic infarcts [99,100]. The midbrain receives its blood supply from the upper BA or PCA, and atherosclerosis in these vessels can lead to midbrain infarctions via perforator occlusion [98]. A study utilizing DWI and MRA revealed that, among 37 patients with pure midbrain infarction, 24 cases were attributed to large artery disease, with SSIs related to PAD being the presumed mechanism in 17 instances [85].

Perforators from the BA supply the anteromedial aspects of the pons [101]. In contrast, PAD-related SSIs frequently extend to the ventral surface, while lesions caused by SVD tend to be smaller and localized in the dorsal region of the pons [101,102]. MRA studies have indicated that BA atherosclerotic stenosis is present in 23% of pontine infarctions [103] and in 39–50% of lesions extending to the basal pial surface [101]. Hence, PAD-related SSIs represent a critical mechanism for pontine infarctions. Notably, bilateral pontine infarctions can lead to severe outcomes, including quadriparesis, gaze palsy, locked-in syndrome, and even death [104]. In many Asian countries, this syndrome is often caused by bilateral multiple branch occlusions associated with extensive BA atherosclerosis. Fisher [46] previously described a patient with locked-in syndrome resulting from bilateral occlusion of the basilar branches due to mural atherothrombosis.

Moreover, PAD-related SSIs can arise from distal VA steno-occlusive lesions, whether due to atherosclerosis or dissection, and are a significant mechanism for stroke in the medulla [105,106,107]. In one study, among 123 patients with lateral medullary infarction studied via angiogram (primarily MRA), VA disease was identified in 83 patients (67%), comprising 5 with proximal lesions, 33 with distal lesions, and 34 with complete VA disease. Thus, 67 patients were classified as having PAD-related SSIs [107]. In another MRA study involving 86 patients with medial medullary infarction, VA atherosclerotic disease relevant to the infarction was identified in 53 patients (62%), with strokes primarily attributed to PAD [86,107]. In a further study of 30 patients with medial medullary infarction, 10 (33%) were found to have relevant VA disease, including seven cases of atherosclerosis and three of dissection [108].

### 2.6. Clinical Issues

As an atherothrombotic condition, PAD typically exhibits more pronounced characteristics of atherosclerosis compared to SVD. A comparison involving 335 SSI patients with PAD and 114 patients with SVD revealed that those with PAD were more frequently associated with atherosclerotic markers, such as coronary heart disease and asymptomatic cerebral artery atherosclerosis. In contrast, the prevalence of SVD markers, including leukoaraiosis and microbleeds, was lower in the PAD group [84]. These differences were also linked to the vascular territory involved; as the territory descended from the MCA to the BA and finally to the VA, markers of atherosclerosis significantly increased while markers of SVD decreased [84]. Another study corroborated the idea that PAD was associated with advancing age and atherosclerotic lesions in other vascular territories [1].

Although the clinical features of SSIs due to PAD and SVD do not fundamentally differ—both result in strictly subcortical infarctions—lesion volumes are generally larger in patients with PAD [90,100]. However, lesion diameter may not necessarily reflect this difference [92], likely due to the occlusion of proximal vessels rather than distal ones. Consequently, the resulting neurological deficits tend to be more severe in PAD patients. Additionally, clinical differences emerge based on the location of the SSI. In the brainstem, motor tracts are situated ventrally while sensory tracts and cranial nerve nuclei are located dorsally. Since PAD-related SSIs are typically found in the ventral part of the brainstem, they more frequently lead to hemiparesis or hemiataxia than to sensory or cranial nerve dysfunction [102].

SSIs can occasionally be associated with neurological progression [109], potentially influenced by factors such as advanced age, diabetes mellitus, elevated C-reactive protein (CRP) levels, and lesion size [110,111,112,113]. However, it remains uncertain whether PAD-related SSIs are more frequently linked to neurological progression than those resulting from SVD. Studies focusing on patients with SSIs in the MCA territory have yielded mixed outcomes; while some suggest that SSIs associated with MCA atherosclerosis are more likely to be linked to neurological progression or an unstable clinical course [114,115], others indicate that neurological progression correlates with subacute DWI lesion volume increases rather than the presence of MCA atherosclerosis [89]. In a study involving 56 patients with acute unilateral pontine infarctions extending to the ventral surface, 22 patients (39%) exhibited PAD-related SSIs [90]. Compared to patients without PAD, those with PAD showed a larger increase in subacute lesion volume on serial DWI. Neurological progression was also more common in the PAD group (36%) compared to the non-PAD group (21%), although this difference did not reach statistical significance. Another investigation of lateral thalamic infarctions found that the presence of PCA atherosclerosis was significantly associated with both increased subacute lesion volume and poor functional outcomes [100]. Collectively, these findings suggest that, at least for patients experiencing posterior circulation strokes, the presence of PAD may be linked to progressive neurological deficits. The increases in lesion volume and neurological progression among patients with PAD-related SSIs may be attributed to either a more extensive atherothrombotic process or a larger area of hypoperfusion in these patients compared to those with SVD [77,78].

Furthermore, patients with PAD-related SSIs were found to experience recurrent strokes more frequently than those with small artery disease [115]. This trend may be associated with the fact that PAD is often linked to older age, coronary heart disease, and other concurrent cerebral atherosclerosis [84]. As a result, the overall clinical outcomes for patients with PAD are likely to be less favorable. Additional prospective studies are necessary to clarify the true impact of PAD on clinical outcomes.

## 3. Neuroimaging Clues

### 3.1. Single Subcortical Infarctions

SSIs represent a significant phenotype of acute ischemic stroke [116]. These infarcts are primarily categorized as lacunar strokes or BOD, depending on their underlying pathophysiological mechanisms [44]. Recent advancements in multi-modal neuroimaging have unveiled various potential mechanisms contributing to subcortical infarcts, including origins from large artery or cardioembolic sources [117]. Differentiating the etiologies of subcortical infarction is vital, as they may require distinct clinical management approaches [84]. However, this differentiation continues to be challenging due to the limited in vivo visualization of perforating arteries.

The current classification system, which is based on infarct size, has limitations in accurately diagnosing subcortical infarcts with respect to their underlying mechanisms [71]. Although high-resolution MRI has improved the visualization of perforating arteries and culpable plaques in subcortical infarcts [118,119], existing imaging modalities still do not permit direct visualization of the responsible perforating arteries. Three-dimensional rotational angiography (3D-RA) offers a valuable tool for effectively visualizing the anatomy and details of perforating arteries, such as lenticulostriate arteries (LSAs) [120,121].

The size of an SSI is one of the factors potentially linked to its etiology, particularly in raising suspicion regarding BOD [122]. Additionally, the etiology is closely tied to prognosis. Although SSIs, also referred to as lacunar infarctions, generally have relatively favorable clinical outcomes, earlier studies indicated that early neurological deterioration (END) occurs in 20–43% of SSI cases, potentially leading to unfavorable functional outcomes [112,123,124]. Predicting whether an acute SSI will deteriorate based on individual patient characteristics and brain imaging data at the time of initial diagnosis is essential for guiding treatment strategies. However, established classifications for SSIs are lacking, and the mechanisms driving END in patients with acute SSIs remain unclear. Various factors have been proposed as potential mechanisms for this progression, including hemodynamic factors, thrombosis extension, excitotoxicity, and inflammation [109,113,125,126,127,128]. As noted previously, SSIs are typically attributed to SVD or stenoocclusion at the orifice of a perforator in the parent artery [129]. BOD presents different arterial pathologies compared to SVD, as atheroma tends to affect vessels more proximally. A recent study found that the incidence of END was significantly higher in subjects with BOD. The most plausible mechanism proposed for this is the occurrence of local thrombosis on the ostial atheroma, alongside thrombus propagation from either proximal to distal or vice versa in the segments of a perforating artery, compounded by the progressive occlusion of lateral branches [128].

A previous study defined “small subcortical infarction” as lesions smaller than 20 mm in transverse diameter within the territory of a penetrating arteriole; however, this definition does not clarify any specific etiopathogenesis [130]. Additionally, prior research has shown that plaques in the middle cerebral artery (MCA) are found in 42% to 60% of patients with lacunar infarction [93,131,132]. Nevertheless, differentiating between BOD and SVD in patients without significant arterial stenosis on MRA remains challenging, and the classification of SSIs based on size is still debated [92,132,133]. Jang et al. [122] identified significant differences in the maximum diameter of the largest lesion on axial views and in the number of slices showing cerebral infarction on transverse planes between patients with and without END. After adjusting for age, hypercholesterolemia, hemoglobin levels, NIHSS scores on admission, and these MRI characteristics, having three or more slices indicating cerebral infarction on a transverse plane emerged as an independent predictor of END in SSIs without relevant artery stenosis (1 vs. 3; OR 14.281; 95% CI 1.76–115.8; *p* = 0.013; 1 vs. 4; OR 14.04; 95% CI 1.65–119.57; *p* = 0.016) [122].

Numerous studies have investigated the association between END and MRI findings in cases of SSI [84,112,116,117,118,119,120,121,122,123,124,126,128,134,135]. Notably, Nakamura et al. [112] found that lacunar stroke patients experiencing END, as indicated by T2-weighted imaging (T2WI), had larger lesions than those without deterioration. Similarly, Terasawa et al. [123] concluded that both the initial infarct volume and its subsequent enlargement contribute to END. Moon et al. [135] demonstrated that an initial infarct size of ≥15 mm was associated with the progression of motor symptoms, while Jeong et al. [136] reported that patients with SSIs characterized by relevant arterial stenosis and branch atheromatous lesions were more likely to experience END. Most previous investigations utilized the classical definition of SSI, which considers stenosis in relevant arteries if it results in ≥50% narrowing of the lumen.

Traditionally, SSIs were classified as a type of SVD, typically caused by lipohyalinosis or fibrinoid necrosis affecting small arteries or arterioles that extend into deeper brain structures [137]. BOD was introduced in 1989 as an alternative mechanism to lipohyalinosis, with lesions generally occurring more proximally along the perforator artery compared to those resulting from lipohyalinosis. Consequently, ischemic lesions associated with BOD may be larger than those related to lacunar infarctions [84]. However, there is considerable variability regarding the dimensional cutoff used to define BOD-related infarcts; in many studies, lesions were identified by a diameter greater than 15 mm on axial DWI [84]. In our research, we found that the visualization of infarcts across three or more consecutive slices on axial DWI images correlated with END in patients whose relevant arteries showed no signs of stenosis on MRA. This suggests that longitudinal damage to small penetrating arteries may contribute to END in patients without significant arterial stenosis, in contrast to those with stenosis. Larger lesions could indicate the presence of a long thrombus, permitting both retrograde and anterograde extension.

### 3.2. Arterial Issues

As previously discussed, perforating arteries are involved in infarctions through two primary mechanisms: branch occlusion disease (BOD) and lipohyalinotic degeneration (LD) [138]. BOD is thought to result from an atheroma in the parent artery, which obstructs the openings of the perforating arteries. This is radiologically characterized by lesions larger than 10 mm in the LSAs and lesions affecting the basal pontine region in the anterior pontine arteries (APA). In contrast, LD pertains to damage to the perforating artery itself [139]. Notably, BOD is more frequently observed in APA infarctions compared to LSA infarctions [140]. Most APA infarctions exhibit atherosclerotic plaques in the basilar artery, which can be visualized through morphological sequences on MRI (and sometimes on CTA), though high-resolution MRI provides better imaging [141].

Arterial tortuosity, which has been linked to hereditary conditions, advanced age, and hypertension [142], can lead to hemodynamic changes that foster the development of atherosclerosis in specific vessels, thereby contributing to BOD [143]. Additionally, arterial tortuosity is closely associated with LD [144].

**Figure 1:**
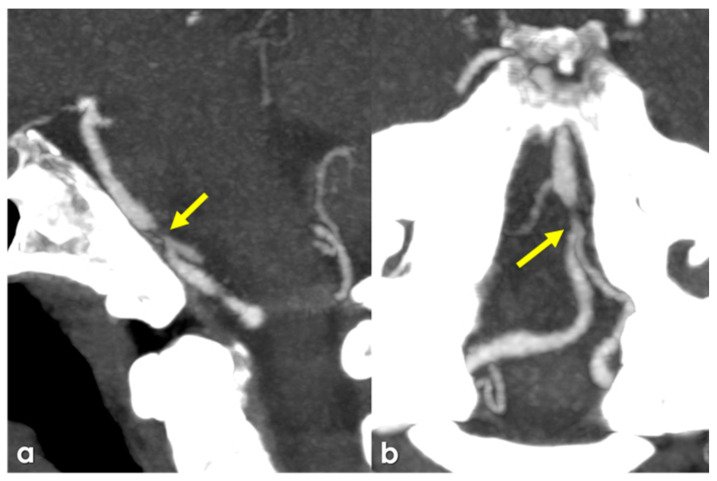
Computed Tomography Angiography (CTA) of a patient with ICAS in the proximal basilar artery (BA), imaged in Multiplanar Reconstruction (MPR) protocol with Maximum Intensity Projections (MPR) processing in sagittal (panel **a**) and coronal (panel **b**) view. The yellow arrows point to the BA stenosis.

**Figure 2:**
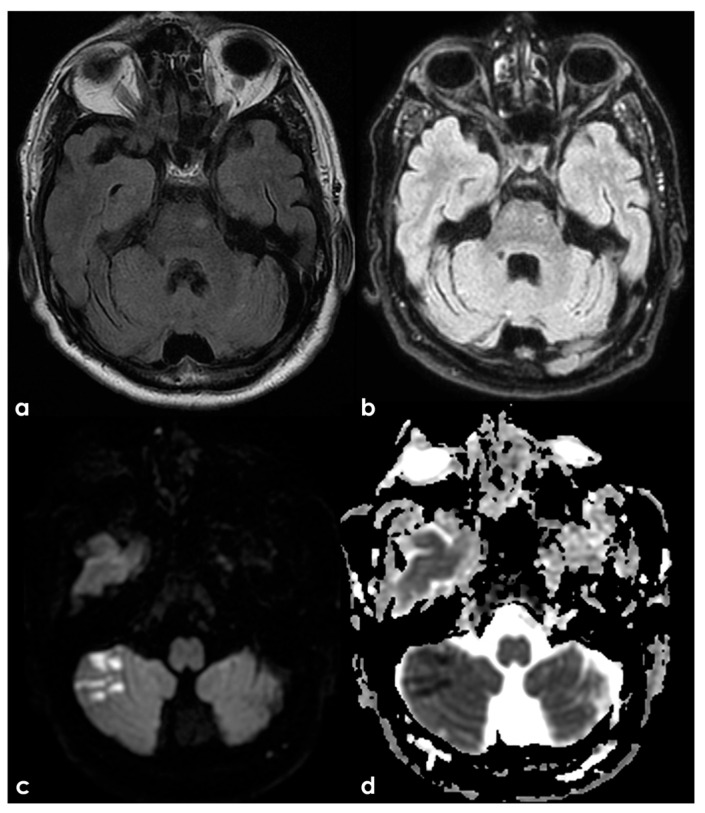
Brain Magnetic Resonance Imaging (MRI) of the same patient referenced in Figure 1. Panel (**a**) and (**b**) show axial Fluid Attenuated Inversion Recovery (FLAIR) sequence ten and thirty days after ischemic stroke, respectively, highlighting a left pontine small subcortical infarction (SSI) (panel (**a**)) that rapidly evolved in a cavitated lesion (panel (**b**)), confirming the proposed timing. In panel (**c**,**d**), Diffusion Weighted Imaging (DWI) and Apparent Diffusion Coefficient (ADC) show a right multifocal cerebellar acute ischemic lesion.

**Figure 3:**
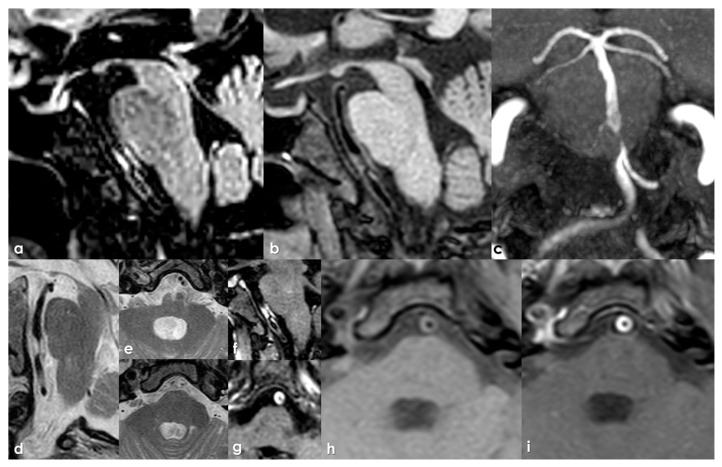
MRI and Magnetic Resonance Angiography (MRA) in the same patient referenced in Figure 1 and Figure 2. Panel (**a**,**b**): sagittal FLAIR at two different time points, respectively, with hyperintense BA walls corresponding with the plaque. Panel (**c**): MRA reconstructed in coronal plane with MIP/MPR protocol, confirming proximal BA stenosis, as in Figure 1 (CTA). Panel (**d**,**e**): sagittal and axial T2 Turbo Spin Echo (TSE) images at the level of the BA. Panel (**f**,**g**): corresponding T1 Dixon sagittal and axial images. Panels (**h**,**i**): magnification of proton density sequence pre- and post-contrast, respectively.

Stenosis in intracranial arteries, such as the MCA, is most effectively detected using noninvasive techniques like MRA and CTA. While digital subtraction angiography (DSA) is considered the gold standard, its invasive nature makes it unsuitable for screening stenosis in patients who lack visual evidence of stenosis in relevant arteries following ischemic events. A previous study demonstrated that MRA is comparable to DSA for identifying stenosis greater than 50%, with sensitivity, specificity, and accuracy rates of 92%, 91%, and 91%, respectively [145]. Figure 1, Figure 2 and Figure 3 show some examples of CTA and MRI/MRA techniques in diagnosing intracranial stenosis.

The vessel wall MRI (VW-MRI) at 3 Tesla was employed to visualize the inner MCA. Chung et al. [131] conducted 3 Tesla VW-MRI scans on 15 patients with acute small subcortical infarctions who exhibited no relevant vessel disease upon MRA (MCA or BA) and identified significant branch atheromatous plaques in nine subjects. This suggests that patients without relevant artery stenosis on MRA may still exhibit features of BOD. A recent study found that the incidence of END was significantly higher in the BOD group [88,122]. However, performing VW-MRI on all subcortical small infarction patients lacking relevant artery stenosis is not feasible.

Using traditional brain imaging techniques, including MRI, MRA, and DWI, the longitudinal length of the infarcted lesion—assessed by its appearance across three consecutive slices on a transverse plane—was significantly associated with the occurrence of END in the above-reported study [131]. Limited studies utilizing modern methods to measure brain perfusion (via computed tomography or MRI) have reported that perfusion deficits correlate with END [146,147,148]. This aligns with the understanding that perforating arteries, known as end arteries, have poor collateral connections and are susceptible to drops in blood pressure. Treatments aimed at enhancing local perfusion, such as induced hypertensive therapy, represent vital management strategies for these patients. One retrospective study indicated that phenylephrine-induced hypertension could lead to early motor recovery without significant side effects in patients with progressing SSIs [149]. Furthermore, a multicenter randomized clinical trial demonstrated that phenylephrine-induced hypertension was safe and resulted in early neurologic improvement and long-term functional independence in patients with non-cardioembolic strokes [150]. Notably, subgroup analyses revealed that treatment-induced hypertension had more pronounced effects on small vessel occlusions compared to larger artery occlusions resulting from strokes [150].

Therefore, the longitudinal length of an infarct lesion in patients with acute SSIs who exhibit no relevant artery stenosis on MRA is a significant risk factor for the development of END, at least in the Korean population [131]. VW-MRI should be considered in patients with SSIs characterized by long longitudinal extension and without relevant artery stenosis to image a non-stenosing plaque.

### 3.3. Perforating Arteries

The LSAs are small perforating arteries that branch from the MCA and ACA to supply essential deep brain structures [151,152]. These arteries are particularly affected by SVD, being responsible for approximately 25% of ischemic (lacunar) strokes, most hemorrhagic strokes, and 45% of dementia cases [153,154,155]. The pathology underlying SVD is not fully understood, and non-invasive imaging of the LSAs could provide critical insights while identifying vascular pathology at an early stage. Cerebral vessels can be visualized using time-of-flight MRA (TOF-MRA), but the small diameters of LSAs (0.10–1.28 mm) [156] make visualization challenging with 1.5T or 3T MRI systems. Ultra-high-field 7T MRI offers enhanced spatial resolution, significantly improving the visibility of LSAs [157,158]. The identification of LSAs can be further enhanced by using gadolinium-based contrast agents [159]. Recent clinical studies have employed 7T TOF-MRA to explore LSA morphology, including aspects like length, branching, and tortuosity [158,159,160,161]. LSA morphology is commonly assessed through coronal maximum-intensity projections (MIPs) of the LSA region [152,159,161,162,163,164,165,166], with some studies focusing solely on the longest LSA branch in each hemisphere [162].

Typically, LSAs are not directly visible using CTA- and MRI-based techniques unless high-field imaging (7T) is utilized [152,159,161,167,168,169]. Catheter angiography remains the most reliable method for imaging LSAs and other major perforating arteries; however, small perforating arteries cannot be visualized effectively with any technique. Koge et al. [170] proposed an image fusion technique combining 3D rotational angiography (3D-RA) and 3D fluid-attenuated inversion recovery (3D-FLAIR) MRI to identify culprit perforating arteries. In a cohort of 118 patients, the culprit perforating artery was successfully identified in 52 patients (44%) [170]. Identification was particularly frequent among younger patients with higher baseline NIHSS scores and a greater prevalence of infarcts in the lentiform nucleus. Among 44 patients with assessable morphology, 61% exhibited stenosis in the proximal segment of the perforating artery. Patients without stenosis had a higher prevalence of atrial fibrillation (AF) compared to those with stenosis (29% vs. 4%) [170]. These findings suggest a link between the morphological features of culprit perforating arteries and the underlying mechanisms of stroke, with stenosis potentially indicating an atherothrombotic etiology in the absence of significant atherosclerosis in the parent artery [170]. A relevant caveat pertains to the inclusion criteria of these studies, mainly involving patients with SSIs of undetermined etiology, whose lesion characteristics deviated from typical SVD (e.g., lesions larger than 20 mm in their largest dimension), or in cases where morphological changes in the parent artery were suspected but inconclusive on MRA or CTA [170]. The morphology and the segment of the parent artery from which the culprit perforating artery originated were also assessed. Parental plaque was defined as any luminal irregularity or stenosis at the orifice of the culprit perforating artery. Parent artery stenosis was observed in 14% of patients without, and in 22% of patients with, a perforating artery stenosis. However, the failure to visualize or the incomplete visualization of small culprit perforating arteries may arise from limitations in resolution, patient motion, or breathing during imaging, as well as inadequate opacification. These perforating arteries might be obstructed by plaques from the parent artery or microemboli, leading to their non-visualization. That said, the origin and morphology of culprit perforating arteries in infarctions located in the lentiform nucleus were effectively visualized, likely due to their larger diameter compared to other perforating arteries [121].

A recent study using VW-MRI uncovered small culprit plaques in otherwise normal parent arteries on DSA in cases of cryptogenic stroke [171]. However, luminal irregularities in patients with proximal stenosis of the culprit perforating artery suggest the presence of a parent artery plaque. These results may illustrate the pathophysiological mechanisms underlying BOD, where plaques in the parent artery obstruct the orifices of the perforating arteries.

VW-MRI has increasingly been employed in diagnosing ICAD and may play a role in differentiating between luminal stenosis and improving the diagnosis of substenotic atherosclerotic plaques. For example, an autopsy study involving 196 cases showed that while intracranial atherosclerosis is typically associated with stenosis, about 20% of cases exhibited advanced atherosclerosis in the absence of significant stenosis [172]. VW-MRI provides an in vivo method for visualizing atherosclerotic plaques, including those that have not resulted in observable stenosis. Furthermore, conventional lumen-based imaging often faces challenges in distinguishing between stenosis caused by atherosclerosis and that caused by vasculitis. This differentiation can be aided by considering the patient’s risk factors and clinical history. Atherosclerosis typically does not affect younger patients and predominantly impacts regions with higher turbulent blood flow, while vasculitis does not demonstrate this tendency [173]. Nevertheless, even with DSA, considerable diagnostic uncertainty remains. Vessel wall imaging shows promise in differentiating vasculitis from ICAD based on patterns of vessel wall enhancement, the extent of persistent enhancement in follow-up imaging, and the involvement of adjacent parenchymal enhancement [174].

VW-MRI requires both a high contrast-to-noise ratio and ahigh spatial resolution to accurately visualize vessel walls and lesions [175]. To achieve this, MRI typically employs field strengths of 3T or higher. The 7T field strength offers an improved contrast-to-noise ratio and more optimal cerebrospinal fluid suppression compared to 3T, enhancing vessel wall visualization, though it is less commonly available [176]. However, some optimized 1.5T equipment can still provide informative imaging, particularly of proximal vessels (BA, VA, ICA, MCA). Compared with 3D sequences, 2D sequences have longer scan times but yield better signal-to-noise ratios and higher image quality. The 3D sequences allow for a more comprehensive evaluation of the vessel and can improve the visualization of small plaque components [177]. A typical VW-MRI protocol includes TOF MRA, 2D and 3D sequences, T1-weighted images with and without contrast, T2-weighted images, and proton density-weighted imaging. Given that VW-MRI is a lengthy procedure, images are susceptible to motion artifacts that can render the study non-diagnostic.

On VW-MRI, a normal vessel should display a uniform concentric thickness, a smooth lumen, and no significant enhancement. A recent meta-analysis evaluated the significance of various imaging features of symptomatic plaques and identified plaque enhancement, positive remodeling, T1 hyperintensity, and surface irregularity as strong biomarkers of symptomatic plaques [178]. While eccentric wall thickening is commonly observed in ICAD [179], the presence of eccentricity was not found to be associated with an increased risk of stroke in one meta-analysis [180]. Abnormal plaque enhancement on VW-MRI is typically defined as enhancement observed on post-contrast T1-weighted imaging that is equal to or greater than the degree of physiological enhancement present in the pituitary gland [181]. Prior studies using extracranial carotid artery specimens obtained from endarterectomy with histopathologically confirmed atherosclerotic disease have shown that enhancement on VW-MRI correlates with neovascularization and inflammation [182]. Plaque enhancement in intracranial vessels has been strongly linked to acute downstream infarcts [180,181]. As the infarct ages, the degree of plaque enhancement gradually decreases [183], and the persistence and degree of enhancement have been associated with stroke recurrence [184]. When interpreting contrast enhancement, it is important to keep in mind that non-pathological enhancement can occur in the walls of the internal carotid artery and vertebral artery as these arteries traverse the dura mater [185]. Intraplaque hemorrhage is another strong indicator of symptomatic plaques and is associated with stroke recurrence [186]. Histopathological studies of extracranial cervical artery specimens have validated that intraplaque hemorrhage correlates with plaques that exhibit high signal intensity on T1-weighted images [187]. Unlike the in vivo validation of plaque characteristics for carotid atherosclerosis due to the availability of samples from carotid endarterectomy, in vivo validation for intracranial arteries remains a challenge. However, caution should be exercised when interpreting VW-MRI results, given the lack of pathological validation for findings in intracranial arteries [188].

**Figure 4:**
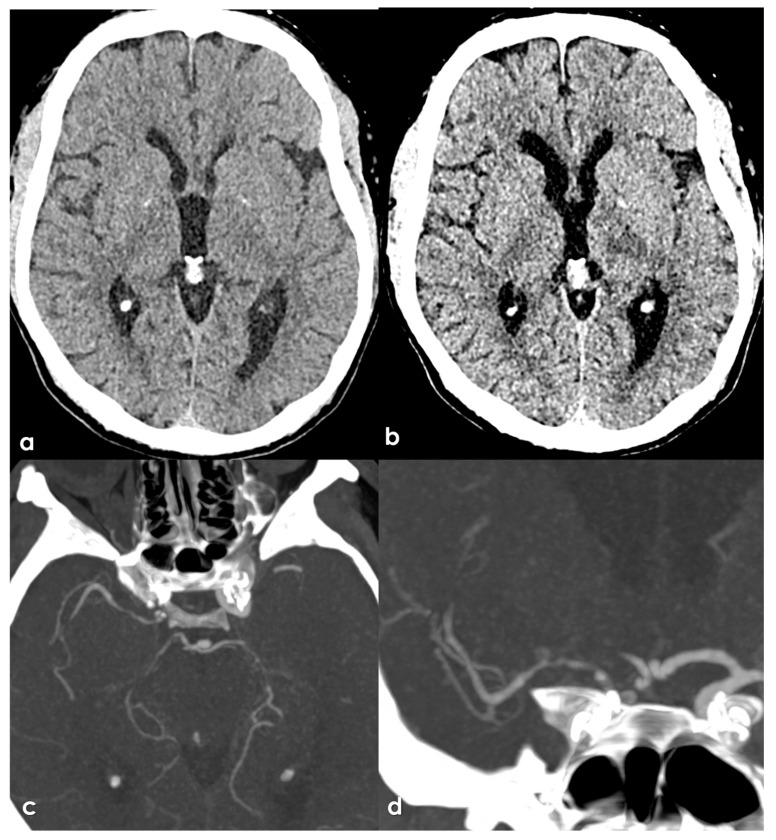
CT-based study of a patient with PAD in the left posterior cerebral artery (PCA) territory and multifocal intracranial stenosis. Panel (**a**,**b**) show non contrast CT scan at the baseline (panel (**a**)) and after 24 h (panel (**b**)), when a recent left capsular ischemic lesion appears. Panels (**c**,**d**) show CTA reconstructed in the axial and coronal plane, respectively, using the MIP/MPR protocol with bilateral ICA stenosis and severe left P2 PCA stenosis.

**Figure 5:**
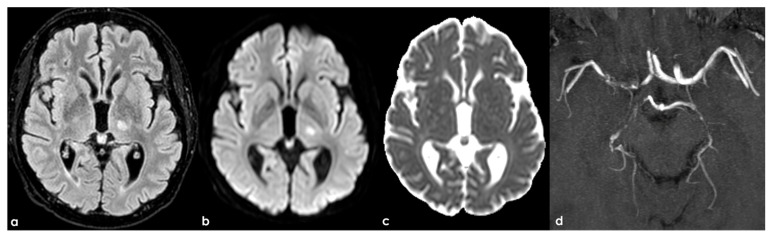
Brain MRI and MRA of the same patient as shown in Figure 4 (panel (**a**), axial FLAIR; panel (**b**), axial DWI; panel (**c**), axial ADC; panel (**d**), MRA in axial plane with MIP/MPR protocol).

Some examples are provided in Figure 4 and Figure 5.

## 4. Medical Therapy

Basically, the medical management of PAD does not differ from the corresponding management of ICAS-related stroke with other imaging patterns (artery-to-artery embolism, hemodynamic lesions, etc.), including an aggressive intervention regarding vascular risk factors and antithrombotic drugs.

Lowering low-density lipoprotein (LDL) cholesterol is a cornerstone in the management of patients with ICAS. The evidence is compelling; multiple studies have shown that elevated LDL cholesterol is a predictor of recurrent ischemic events. For instance, a prospective observational study involving 74 patients highlighted that elevated LDL at the time of the index stroke significantly predicted recurrent infarct at 6 to 8 weeks post-enrollment [189]. Furthermore, the SAMMPRIS trial demonstrated that higher LDL and non-high-density lipoprotein levels during follow-up were associated with a greater likelihood of vascular events at the three-year mark, revealing an odds ratio (OR) of 1.2 for a 10 mg/dL increase in LDL [190]. In response to these findings, high-intensity statins, such as atorvastatin and rosuvastatin, are recommended as the first-line agents for LDL reduction in patients with ICAS [191,192]. The Treat Stroke to Target trial, which compared different LDL targets, found that patients whose LDL was targeted below 70 mg/dL experienced significantly lower rates of major cardiovascular events, including strokes [193]. High-intensity statin therapy can lead to plaque regression over time, thus stabilizing symptomatic plaques [194,195]. For patients unable to meet LDL targets with statins alone, the addition of ezetimibe is recommended to further lower LDL levels [191,192,196]. Notably, a subgroup analysis from the IMPROVE-IT trial reported that ezetimibe plus simvastatin was superior to simvastatin alone in reducing recurrent ischemic strokes [197]. The use of PCSK9 inhibitors and bempedoic acid is gaining traction as adjunct therapies for patients who struggle to achieve LDL targets [198,199].

The management of hypertension is equally critical in reducing the risk of recurrent strokes in ICAS patients. Maintaining blood pressure below 140/90 mm Hg significantly reduces the risk of vascular events [191,192]. A post hoc analysis of the WASID trial indicated that higher blood pressure is correlated with increased rates of ischemic strokes and vascular events [200]. Similarly, the MyRIAD trial demonstrated a trend toward increased risk for recurrent strokes associated with elevated blood pressure during follow-up [201]. While more aggressive blood pressure targets (e.g., <130 mm Hg) are recommended for patients with ischemic strokes from other causes, the evidence for ICAS remains inconclusive. In fact, a trial involving 132 patients found that those with a more aggressive target exhibited larger infarct volumes, suggesting caution in aggressively lowering blood pressure in this population [202]. Specific antihypertensive medications, such as thiazide diuretics, angiotensin-converting enzyme inhibitors, or angiotensin II receptor blockers, are recommended to achieve these targets [191,203].

Diabetes is another major risk factor linked to ICAS, with studies indicating that patients with diabetes are at a higher risk for recurrent strokes [67,190,204,205,206,207,208]. However, compelling evidence showing that improved glycemic control directly correlates with better outcomes in ICAS patients is lacking. A retrospective study using VW-MRI found that higher hemoglobin A1C levels were independently associated with the risk of recurrent ischemic strokes [209]. Diabetes management should aim for a hemoglobin A1C level of ≤7%, although treatment should be individualized based on patient circumstances.

The SAMMPRIS trial found that physical inactivity increased the risk of recurrent stroke, myocardial infarction, or vascular death by five-fold, making it the strongest risk factor correlated with recurrent strokes [190]. The authors advocate for a minimum of 10 min of moderate-intensity aerobic activity four times a week or 20 min of vigorous-intensity aerobic activity twice a week [190].

Smoking is a well-established risk factor for cardiovascular events, including ischemic stroke [210,211,212]. Although there is no definitive evidence proving that smoking cessation reduces the risk of recurrent strokes in ICAS, counseling for smoking cessation remains a vital component of secondary prevention for all ischemic stroke patients who smoke [191].

Antiplatelet therapy has been the mainstay of treatment for ICAS since the WASID trial, which demonstrated that aspirin is effective in reducing ischemic stroke rates [213]. The trial compared aspirin to warfarin and showed that the warfarin group experienced higher rates of all-cause death and major hemorrhage (4.3% vs. 9.7% and 3.2% vs. 8.3%, respectively) [213]. Consequently, aspirin became the preferred antithrombotic treatment for ICAS. Following the SAMMPRIS trial, dual antiplatelet therapy with aspirin and clopidogrel emerged as a preferred treatment for patients with severe ICAS (70–99% stenosis). This was largely due to post hoc analyses that indicated fewer vascular events with short-term dual therapy compared to traditional aspirin therapy (12.2% in SAMMPRIS vs. 25% in WASID at one year) [11,214]. Although the current guidelines recommend a duration of up to 90 days, some studies suggest that the high risk of ischemic stroke persists beyond this period, warranting consideration of longer therapy [215].

In summary, the management of symptomatic intracranial atherosclerotic stenosis necessitates a comprehensive, target-driven approach focusing on risk factor control, lifestyle modifications, and the appropriate use of antithrombotic therapies. Ongoing studies, particularly those investigating the role of direct oral anticoagulants and the optimal duration of antiplatelet therapy, may further refine treatment strategies and improve outcomes for patients suffering from this high-risk condition.

## 5. Impact on Endovascular Procedures

Angioplasty or stenting of intracranial atherosclerotic stenosis is technically feasible but carries a higher risk of peri-procedural complications, including arterial thrombosis, distal embolization, vessel dissection, vessel rupture, vasospasm, and perforator stroke (PS) [215,216,217,218,219]. A significant concern in performing angioplasty or stenting for intracranial atherosclerosis is the postprocedural patency of side branches, particularly the perforating arteries, even during endovascular treatment for aneurysms [220,221,222]. Compromise regarding these perforating vessels and arterial dissection are associated with PS following intracranial angioplasty. Furthermore, stent struts that cross the ostia of perforators in the atherosclerotic vessel may lead to occlusion of these vessels after stenting [220]. Due to their small diameter, perforating arteries are often poorly visible on standard imaging studies, yet they are crucial for supplying essential neural structures, such as the internal capsule and hypothalamus. Complications associated with perforating artery infarction can significantly delay or prevent recovery after an intracranial intervention.

The quality of reporting of these complications is generally low across studies. A notable investigation assessed the frequency, clinical course, and functional outcomes of PS resulting from the elective stenting of symptomatic intracranial stenosis in an Asian population, involving 169 consecutive patients with 181 symptomatic intracranial stenoses who underwent stenting procedures [223]. The frequency of PS was found to be 3.0% (5/169 patients). Patients with preoperative perforator infarcts adjacent to the stenotic segment (PIAS) identified on MRI exhibited a higher frequency of PS and exacerbation following intracranial stenting (8.2%, 4/49) compared to those without preoperative PIAS (0.8%, 1/120; *p* = 0.031). Four PS incidents occurred during the procedure, while one occurred 10 h after stenting. The authors concluded that patients with preoperative perforator infarcts adjacent to the stenotic segment have an increased risk of PS after elective stenting.

The Stenting and Aggressive Medical Management for Preventing Recurrent Stroke in Intracranial Stenosis (SAMMPRIS) trial [224] was a randomized, multicenter clinical trial funded by the National Institute of Neurological Disorders and Stroke. Early results revealed that by 30 days, 14.7% of patients in the stenting group and 5.8% in the medical management group experienced either death or stroke. Long-term outcomes from this trial indicated that aggressive medical treatment was superior to percutaneous transluminal angioplasty and stenting (PTAS), with the Wingspan stent in patients with recent transient ischemic attacks or strokes attributed to 70–99% atherosclerotic intracranial arterial stenosis, particularly in high-risk patients. Subgroup analysis in the SAMMPRIS study indicated that infarctions involving perforator territories constituted the majority (71.4%) of ischemic events. A buildup of atheromatous plaque in the parent artery has been identified as an independent predictor of branch occlusion following stenting [223,225]. This branch occlusion mechanism has primarily been attributed to plaque shift after stenting, often referred to as the “snow plow effect” [225]. However, the SAMMPRIS trial did not report any new perforator territory infarctions in patients with a recent perforator stroke who received the Wingspan stent, despite 21% qualifying with perforator strokes [224].

Alternative mechanisms for perforator occlusion may exist beyond plaque shift. Simulated biomechanical stress using the finite element method has been used to demonstrate the interaction between stents and arteries [226,227]. In a model utilizing the Wingspan stent from the SAMMPRIS study [224], the orifice of the perforator was shown to stretch in a circumferential direction, indicating that structural deformation could contribute to perforator occlusion post-stenting [228]. The study simulated biomechanical stress distributions in an atherosclerotic model with structural deformation. Atheromatous plaques typically develop along the outer walls of arterial branches and curvatures, where local flow disturbances lead to low or oscillatory shear stress [229]. For the MCA, asymmetric plaque was predominantly detected on the ventral and inferior walls opposite the perforator orifice [230]. Notably, the orifice of the perforator experienced circumferential stretching after stenting, corresponding to areas with high circumferential stress (CS) values. CS has been reported to peak between stent struts and is proportional to the gradient of environmental radial stress (RS) values [227]. Elevated RS values can amplify the stretching effect, alongside high CS. The phenomenon of stretched branch orifices leading to branch occlusion has been observed after stenting in mild atherosclerotic lesions with 25% stenosis [220]. An elongated orifice can disrupt physiological hemodynamics, potentially leading to thrombus formation and reduced blood flow. Even a minor elliptical change can significantly alter wall shear stress around the circumference of a cerebral artery [25]. In particular, an atherosclerotic perforator with impaired blood flow may swiftly result in an ischemic infarction after stenting. Research has indicated that a high balloon–artery ratio correlates with branch occlusion [231]. Balloon-expandable stents, which exert more stress on arterial walls, may lead to more frequent perforator occlusions than self-expanding stents [232]. The stretching effect, combined with excessive stress concentration, may elucidate the mechanism of perforator occlusion following Wingspan stenting.

In this asymmetric plaque model, plaque shift over the orifice was not observed. If there is a plaque surrounding the perforator orifice in clinical practice, pre-ballooning or stenting may provoke plaque shift or rupture, leading to perforator occlusion. Micro-embolism after plaque rupture is another potential cause of perforator occlusion. Furthermore, stent struts positioned over the orifice may obstruct flow to the perforator or induce thrombus formation. The metal coverage and endothelization of stent struts have been postulated as factors [233]. However, the lower metal coverage of the Wingspan stent compared to a flow-diverting stent makes it less likely to occlude the branch [234]. Another investigation examined the impact of covering the origins of perforating arteries with stent struts using two geometric models, assessing the coverage of perforating artery ostia from a collection of 3D models of twenty-three MCAs and ten BAs [235]. Flow-diverting stents reduced the area of the perforator origin by 20–30%, while conventional stents covered at least one-third of the origin in nearly every second perforator studied. Smaller perforating arteries may be completely occluded by stent struts. When employing a telescopic technique with two stents, the coverage percentages were approximately twice as high. Stenting can significantly decrease the effective cross-sectional area of the perforating artery origin. The degree of influence varies with the type of stent: flow-diverting stents exert a mesh effect, whereas classic stents are related to strut width. The effect is exacerbated by the use of two stents. Considering the supply areas of the pontine and LSAs, even small perforating artery infarctions can lead to severe neurological complications. Therefore, to enhance the safety of intracranial artery stenting, it is prudent to design and utilize stents with the narrowest possible struts.

In general, the degree of stenosis caused by the coverage of ostia is contingent on the size of the perforator, and for small LSAs, it can completely cover the ostium. Although small perforators supply smaller volumes of nerve tissue, resulting in smaller lacunes, they can still hold clinical significance due to the critical areas they supply, including the internal capsule and thalamus. The impact of flow-diverting stents on the patency of perforating arteries is primarily influenced by the mesh effect rather than the effect of a single stent strut. In contrast, stents with wider struts (100–150 μm) serve different functions and indications. These struts form a less dense network, primarily aimed at supporting embolization material in aneurysms. The size of the struts is comparable to the width of the perforator ostia, resulting in at least one-third of the origin of every second perforator being covered. With the telescopic technique, this effect is further pronounced. The sparser strut network decreases the likelihood of a stent element covering a perforating artery origin, but when it does occur, the impact is more considerable than with flow diverters; when using a stent with a strut width of 150 μm, up to 50% of every fourth LSA origin may be covered. Because the locations of LSA origins vary, analyzing the microanatomy and planning the procedure is crucial, as this may allow for the avoidance of ostial coverage.

A similar issue has been addressed outside BOD-related stroke, i.e., in flow-diverter implantation, where both early and late ischemic complications are documented in the literature [58,236,237,238,239], with the percentage of acute perforating artery strokes being notably higher in the vertebrobasilar system, reaching 7% [240,241]. This rate is similar to the one occurring after stenting for intracranial artery stenosis with conventional stents, ranging from 2.5% to 10.3% [242,243,244]. The issue of side branch patency is also a concern in interventional cardiology, although of minor relevance [245,246,247]. Furthermore, technical aspects of neuroradiological interventions—such as oversizing, undersizing, and overlapping stents—may further compromise inflow to perforating arteries [248,249]. Regarding late ischemic complications, the effect of stenting on the remodeling of perforator ostia requires further investigation [18,25,250,251]. Clinically, it is important to remember that the mechanical effects analyzed in our study may coexist with additional issues, such as atherosclerotic plaque rupture during the stenting of stenosis or thrombosis following flow diversion for fusiform aneurysms. The clinical significance of potential ischemic complications is determined more by the areas supplied by the perforator than their diameter. For example, pontine arteries (branches of the BA) supply several vital neural structures, meaning that pontine artery infarctions are rarely clinically silent and are typically associated with cranial nerve palsy and contralateral hemiplegia [252]. Conversely, lenticulostriate infarctions may go unrecognized if the supplied area does not involve eloquent structures [21]. Thus, to accurately determine the incidence of ischemic complications following intracranial artery stenting, routine brain MRI—particularly diffusion-weighted imaging (DWI) and apparent diffusion coefficient (ADC) sequences—should be conducted, not only when new neurological symptoms develop [18,253]. Given the geometric model results, it seems prudent to develop and utilize stents with narrower struts. Recent studies indicate that chronic hypoperfusion is sufficient to contribute to the development of dementia [244,254,255,256]. Investigating the effect of impaired blood flow through perforating arteries after stenting on brain aging and cognitive performance would be beneficial. This issue is particularly relevant in light of recent reports indicating that the origins of deep-perforating arteries may be narrowed by intimal hyperplasia, with the degree of stenosis potentially increasing with age [257]. Consequently, stenting may further disrupt blood supply to the basal ganglia and exacerbate hemodynamic changes, promoting brain aging.

The limitations of in vitro models [227] include the absence of hemodynamic analysis, simulations of parent artery deformation, and oversimplified assumptions. In fact, their calculations presuppose that the stent strut follows the long axis of the perforating artery ostium, but other configurations are possible, both more (ostium covered obliquely or not at all) and less (ostium covered by the junction of stent struts) favorable. However, the impact of intracranial artery stenting on local hemodynamics and the remodeling of perforating arteries, alongside chronic hypoperfusion and cognitive performance, requires clarification. In addition, these findings support the design of stents with narrower struts, considering the balance between the risk of ischemic complications and the fulfillment of the intended function.

### Focus on Basilar Artery

The most prevalent complication of endovascular therapy is perforating artery occlusion, with stenting for basilar artery (BA) atherosclerotic stenosis linked to a stroke or mortality rate as high as 21.6% [258]. In cases of coronary artery and MCA atherosclerosis, plaques tend to form opposite the openings of branch or perforating arteries. When plaques are situated near these openings, complications are more likely to arise during stent implantation, a phenomenon often referred to as the “snow shovel” effect during the procedure [23,259]. The distribution of basilar atherosclerotic plaques typically affects all arterial walls—ventral, dorsal, and lateral—where plaques on the dorsal and lateral walls are associated with symptomatic pontine infarctions, rather than asymptomatic ones [260]. VW-MRI can be employed to assess the location vulnerability of plaque, and its relationship to significant branch vessels in cases of severe stenosis of the symptomatic basilar artery, enhancing the safety of endovascular procedures [261,262].

A single-center study conducted on an Asian population aimed to utilize preoperative VW-MRI and DWI, combined with the CISS typing system [263]. In this selected population, according to the CISS system [68], the pathogenesis involved carrier artery plaques blocking the perforating arteries (termed the perforation group), while patients with low perfusion or emboli clearance showed lesions primarily located in arterial branches distant from the basilar artery stenosis. The mean age of the 20 patients in the perforation group was 62.00 ± 8.27 years, compared to 59.67 ± 6.83 years in the hypoperfusion group (27 patients). Balloon dilation was performed in 22 cases, while balloon dilation combined with stenting was performed in 25 cases. Notably, there were no complications, such as basilar artery perforation, stent thrombosis, or cerebral hemorrhage, post-surgery. Two instances of arterial dissection-like manifestations were observed after balloon dilation, but these resolved after stent implantation without significant postoperative symptoms. Four patients in the perforation group exhibited increased NIHSS scores, with new DWI lesions indicating new perforating infarctions in the pontine region. New-onset ischemic strokes were observed 7 days post-surgery, with 20.0% in the perforation group compared to 0.0% in the hypoperfusion group, a statistically significant difference (*p* = 0.027). In the binary logistic regression model, age and a history of hypertension were significantly associated with the incidence of ischemic strokes at 7 days, with a significant association found between the perforation and hypoperfusion groups (*p* = 0.003, OR = 2.347; 95% CI = 2.056–4.268). The proportion of ventral plaques (74.1%) in the hypoperfusion group was significantly higher than in the perforation group (15.0%), while the proportion of dorsal plaques (33.3%) was lower than in the perforation group (90.0%), with significant statistical differences (χ^2^ = 16.045, *p* < 0.001; χ^2^ = 15.092, *p* < 0.001). The findings from the proposed study [263] suggest that the risk of ischemic stroke is greater in patients with perforator artery obstruction undergoing endovascular therapy, whereas patients with low perfusion or embolus removal face a lower risk. Early results from the SAMMPRIS trial indicated that by 30 days, 14.7% of patients in the stenting group had either died or suffered a stroke [264]. In the Vitesse Intracranial Stent Study for Ischemic Stroke Therapy (VISSIT) trials, the 30-day safety endpoint for any stroke or hard TIA within 30 days was 24.1% in the stent group [38]. In contrast, Miao et al. reported only 4.3% of patients experiencing TIA or stroke, with just seven (2.3%) having ischemic stroke complications [265]. One key difference is that the inclusion criteria for the SAMMPRIS and VISSIT trials were broader, while the patients in the Miao study primarily had hypoperfusion etiologies, effectively excluding those with perforating artery strokes. A Chinese study on stent treatment for symptomatic intracranial vertebrobasilar artery stenosis reported seven ischemic events within 24 h post-surgery, including one TIA and six strokes (7.2%, 7/97), all attributed to perforating artery damage [266]. An analysis of perioperative complications in the SAMMPRIS trial indicated that perforating infarction was the most common complication following balloon dilation and stenting of symptomatic BA stenosis [58]. Patients with basilar artery stenosis and pontine infarction are particularly susceptible to postoperative complications due to perforating artery occlusion, which can result from plaque dissection, stent coverage, vascular straightening or curvature, thromboembolism, and other factors. Thus, intraoperative avoidance of perforating artery injury is crucial for the success of endovascular therapy.

Currently, digital subtraction angiography (DSA) is regarded as the “gold standard” for evaluating the degree of luminal stenosis in arteries, primarily showing the location and extent of such stenosis [267]. However, the success of treating intracranial atherosclerotic stenosis extends beyond this, as the relationship between atherosclerotic plaque positioning and perforating vessels is also a critical factor influencing postoperative complications [268]. High-resolution magnetic resonance imaging can assess plaque stability, location, and relationship with perforating vessels by indicating the degree of plaque vulnerability [269,270]. The distribution of atherosclerotic plaques in the symptomatic middle cerebral artery predominantly affects the ventral wall, with the upper and dorsal walls being less affected [271,272]. This distribution may be due to vascular endothelial damage from hypertensive shock, a primary cause of atherosclerosis. In the MCA, the ventral wall experiences the most pressure from blood flow, making it a common site of plaque formation. Understanding the distribution of arterial plaques provides insight into protecting perforating vessels during endovascular treatment.

In patients with MCA atherosclerosis, plaques tend to form opposite the openings of branch or perforating arteries. When plaques are located near these openings, complications are more likely during endovascular treatment, again associated with the “snow shovel” effect during balloon or stent implantation [265,273]. The pontine branches of the basilar artery originate from both sides and the back, numbering around a dozen, with varying lengths. In cases of basilar atherosclerotic stenosis, if plaques are near the perforating artery orifice, the squeezing process of plaques during endovascular therapy, balloon dilation, or stent deployment may block the pontine perforating vessels, leading to severe complications. Miao et al. [265] effectively excluded patients with perforating artery strokes, thereby minimizing the risk of exacerbation due to endovascular therapy.

Currently, the preoperative evaluation for endovascular treatment of basilar atherosclerotic stenosis primarily relies on imaging examinations, with limited integration of the pathogenesis of symptomatic cerebral infarction. In the Chinese study [263], patients were classified into perforation and hypoperfusion groups based on the CISS classification system. The results showed 23 cases of ventral plaque, 24 cases of left plaque, 30 cases of right plaque, and 27 cases of dorsal plaque across the two groups. The hypoperfusion group exhibited a higher proportion of ventral plaques and a lower proportion of dorsal plaques, while no significant difference was noted in the left and right plaque proportions between the groups, consistent with previous findings [65]. Coupling these results with the incidence of ischemic stroke at 7 days post-endovascular treatment, the study suggests that the incidence of ischemic stroke in the hypoperfusion group is lower than in the perforation group. This is likely due to ventral plaques being predominantly located at the basilar artery stenosis in the hypoperfusion group, while the pontine branches generally emerge from the dorsal, left, and right sides of the basilar artery, leading to a higher risk of occlusion when plaques are present in the perforation group. This observation is supported by studies [265,267,274,275,276,277,278,279,280] like that of Abe A [277], which suggest that stent plastic can produce a “snow shovel” effect, where intraoperative balloons or stents may push atheromatous material near the perforating artery opening into the perforating vessel, causing occlusion.

## 6. Conclusions

This comprehensive review emphasizes the critical role of PAD in the context of ischemic strokes, particularly regarding the implications for endovascular treatment. The following key conclusions can be drawn from the analysis:

1. Recognition of PAD’s Impact: PAD is a significant contributor to ischemic strokes, particularly in patients with underlying intracranial atherosclerosis. Its presence often complicates the clinical picture, leading to larger lesions and more severe neurological deficits compared to small vessel disease (SVD). Clinicians must recognize PAD as a distinct entity that warrants specific attention in stroke assessment and management.
2. Endovascular Treatment Challenges: Interventions such as angioplasty and stenting present unique challenges when treating patients with PAD. The review highlights a higher incidence of complications, including perforating artery occlusion, which can lead to significant morbidity and mortality. The mechanisms underlying these complications, such as the “snow shovel effect,” must be acknowledged to mitigate risks during procedures.
3. Importance of Advanced Imaging Techniques: Traditional imaging methods may fail to adequately identify the presence of PAD, particularly in cases with non-stenosing plaques. The review advocates for the adoption of advanced imaging modalities, such as high-resolution MRI, which can improve the visualization of atherosclerotic plaques and assist in more accurate diagnoses. This enhanced diagnostic capability is crucial for informing treatment strategies and optimizing patient outcomes.
4. Need for Tailored Treatment Strategies: Given the complexities associated with PAD, there is a pressing need for individualized treatment approaches. Strategies that consider the specific characteristics of the atherosclerotic plaques, the anatomical relationships of the perforating arteries, and the hemodynamic implications are essential for successful endovascular interventions.
5. Future Research Directions: The review identifies critical gaps in the current literature regarding PAD and endovascular treatment. Future studies should focus on establishing standardized protocols for the diagnosis and management of PAD, particularly in diverse populations. Additionally, prospective clinical trials are needed to evaluate the efficacy of new stenting technologies and techniques designed to minimize complications related to perforating artery occlusion.
6. Clinical Practice Implications: The findings of this review underscore the necessity for a paradigm shift in clinical practice. Stroke specialists should be equipped with the knowledge of PAD’s implications, ensuring that comprehensive assessments include consideration of parent artery disease. This approach will enhance decision-making processes and improve the overall management of patients at risk for ischemic strokes.

In conclusion, addressing the multifaceted challenges posed by parent artery disease is essential for improving outcomes in patients with ischemic strokes. By enhancing diagnostic capabilities and tailoring treatment strategies, clinicians can better navigate the complexities of PAD, ultimately leading to more effective stroke management and reduced recurrence rates.

## Data Availability

No new data were produced in this paper.
